# Hippo pathway elements Co-localize with Occludin: A possible sensor system in pancreatic epithelial cells 

**DOI:** 10.1080/21688370.2015.1037948

**Published:** 2015-05-01

**Authors:** Ana Santos Cravo, Edward Carter, Mert Erkan, Emma Harvey, Makoto Furutani-Seiki, Randall Mrsny

**Affiliations:** 1 Department of Pharmacy and Pharmacology; University of Bath; Bath, UK; 2 Department of Surgery; Koc University School of Medicine; Istanbul, Turkey; 3 Department of Biology and Biochemistry; University of Bath; Bath, UK

**Keywords:** dobutamine, occludin, pancreatic cancer cells, tight junctions, YAP

## Abstract

External adherens junction-based cell-cell contacts involving E-cadherin interactions function to sense planar cell status and modulate epithelial cell proliferation through Hippo (Hpo) and non-canonical Wnt pathways signaling. We hypothesized these regulatory processes should also be sensitive to a similar cell-cell contact sensor associated with apical-basal polarity events at epithelial surfaces. We used 2 human pancreatic cancer cell lines to explore this hypothesis: one with the capacity to form functional tight junction structures and polarize (HPAFII) and one lacking this capacity (AsPc1). Occludin (Ocln), a tetraspanning protein associated with TJ structures and capable of establishing external cell-cell contacts, was observed to partially co-localize with Hpo elements YAP (c-yes associated protein) and TEAD (TEA-dependent), which function to drive a proliferative transcription program. Treatment with dobutamine, known to affect YAP, was shown to suppress proliferation in an Ocln-dependent manner. Blockade of protein kinase C-zeta (PKC-ζ) diminished transepithelial electrical resistance (TER) of HPAFII monolayers that was not corrected by dobutamine treatment while the loss of TER resulting from inhibition of ROCK1 could be partially recovered. Examination of normal and cancerous human pancreatic biopsies showed that the cellular localization of Ocln, c-Yes, YAP, and TEAD were similar to HPAFII for normal cells and AsPc1 for cancerous cells. Together, these results suggest a link between Hpo and signals emanating from cell-cell contacts involving Ocln that may regulate pancreatic cell proliferation through the coordination of planar cell polarity with apical-basal polarity events.

## Abbreviations

AJ: adherens junctions
APC: apical polarity complex
Dob: Dobutamine
EMT: Epithelial Mesenchymal Transition
HDAC: histone deacetylase
Hpo: Hippo
HRP: Horseradish peroxidase
Ocln: Occludin
PDAC: Pancreatic Ductal Adenocarcinoma
PKCζ-PS: PKCζ Pseudosubstrate
PKC-ζ-PS: protein kinase C zeta pseudosubstrate
TEAD: TEA-domain family member
TER: transepithelial electrical resistance
TJ: Tight junction
Tric: Tricellulin
YAP: c-Yes associated protein
ZO-2: zonula occludens-2

## Introduction

The Hippo (Hpo) tumor suppressor pathway plays a critical role in controlling organ growth, stem cell function, regeneration, and tumor suppression (Johnson and Halder, 2014). Disrupted regulation of Hpo pathway elements, in particular YAP, correlates with the loss of epithelial cell lateral crowding control that is observed as unregulated proliferation in the context of cancer (Pan, 2010; Poon et al., 2011). Translocation of YAP from the nucleus to the cytoplasm with its subsequent phosphorylation and degradation is correlated with suppression of cell proliferation (Pan, 2010). Regulatory factors including β-catenin, the protein phosphatase PP2A, and the scaffolding protein 14-3-3 have been implicated in processes that suppress cell proliferation through adherens junction (AJ) contacts and Hpo signaling, providing a mechanism of proliferation control based upon lateral cell density within an epithelium (Schlegelmilch et al., 2011). This mechanism links AJ regulatory elements to YAP de-phosphorylation events that control its nuclear localization, where it combines with transcription factors such as TEAD to drive the production of a proliferative program involving connective tissue growth factor (CTGF). Thus, E-cadherin localized at AJ structures provides an extracellular cell-cell contact sensor to regulate nuclear YAP localization and suppress epithelial cell proliferation at high lateral cell density.

Epithelial barriers must not only regulate cell-cell planar polarity to restrict lateral growth, they must also coordinate proliferation with apical to basal polarity. Events involving the disorganization and/or dysfunction of TJ components have been correlated with hyper-proliferative responses in the context of inflammation and cancer (Bruewer et al., 2006; Edelblum and Turner, 2009; Martin et al., 2010; Tobioka et al., 2004a; Tobioka et al., 2004b). Previous studies have shown that cytoplasmic components of the TJ, such as zonula occludens-2 (ZO-2), participate in the nuclear localization of YAP in a process that is independent of its phosphorylation state (Spadaro et al., 2012). To date, no TJ-associated element has been identified that might function as a sensor for apical to basal polarity status in a manner similar to the role played by E-cadherin in AJ structures for lateral cell density (Harvey et al., 2013). We conjectured that Hpo pathway signaling could also be involved in sensing TJ status, and thus apical to basal polarity, through a protein sensor that forms cell-cell contacts in association with these structures. Indeed, an extracellular sensor for detection of TJ elements has been previously hypothesized (Farkas, et al., 2012). Our studies suggest the TJ protein occludin (Ocln) as a candidate cell-cell sensor that can fine-tune the suppression of proliferation established by AJs.

Although Ocln was the first transmembrane TJ protein identified (Furuse et al., 1993), its role in epithelial cell function is still unclear as it is not required for TJ formation; mice lacking Ocln can produce claudin-based TJs that provide essential barrier function, but these animals exhibit a complex phenotype that suggests a role for this protein in the functional properties of cell-cell interactions (Saitou et al., 2000). Loss of Ocln expression or disruption of its normal cellular organization is associated with inflammatory events (Bruewer et al., 2006; Edelblum and Turner, 2009) and epithelial cell transformation (Sawada, 2013), both of which involve hyper-proliferation. Interestingly, the coiled coil domain of Ocln interacts selectively with PKC-ζ and the non-tyrosine kinase c-Yes (Chen et al., 2002a). PKC-ζ is important for apical to basal polarization events and the c-Yes interaction with Ocln appears to be essential for TJ formation (Chen et al., 2002a; Nusrat et al., 2000). As c-Yes is a *bona fide* binding partner of YAP (Aragona et al., 2013), we examined whether Ocln could function as a potential sensor to regulate proliferation signals involved in apical-basal epithelial cell polarity. We tested this hypothesis using 2 human pancreatic cancer cells lines with different potentials to polarize *in vitro*: HPAFII and AsPc1 that form robust and compromised TJ barriers, respectively.

We observed partial intracellular co-localizations between Ocln and c-Yes, YAP, and TEAD that were consistent with multiple, dynamic associations dependent upon cell-cell interactions. Manipulation of Ocln expression could be achieved by treatment with dobutamine, a small molecule known to induce YAP translocation from the nucleus to the cytoplasm through a mechanism independent of Hpo signaling (Bao et al., 2011). Further, dobutamine treatment suppressed cell proliferation of HPAFII and AsPc1 cells in a dose-dependent and Ocln-dependent manner; events associated with reduced nuclear localization of YAP and TEAD. Decreased TJ integrity through the actions a Rho-associated protein kinase (ROCK) inhibitor could be partially corrected by dobutamine to suppress cell proliferation in an Ocln-dependent fashion. Enhanced cell proliferation resulting from the disruption of TJ function using a PKC-ζ inhibitor failed to be affected by dobutamine treatment. Finally, we examined Ocln, c-Yes, YAP, and TEAD cellular distribution in normal and pancreatic cancer biopsies; these results corroborated findings made for the non-proliferative and proliferative formats identified in the HPAFII and AsPc1 cell lines, respectively. Together, our results suggest that Ocln has the characteristics required to function as a sensor of apical to basal polarity events through its ability to co-localize Hpo signaling elements to forming TJ structures and thus provides a potential mechanism to coordinate these processes with AJ-mediated regulation of lateral growth in an epithelial tissue barrier.

## Results

### Assessment of Ocln with Hpo pathway elements in cells capable of polarization

**Figure 1.**
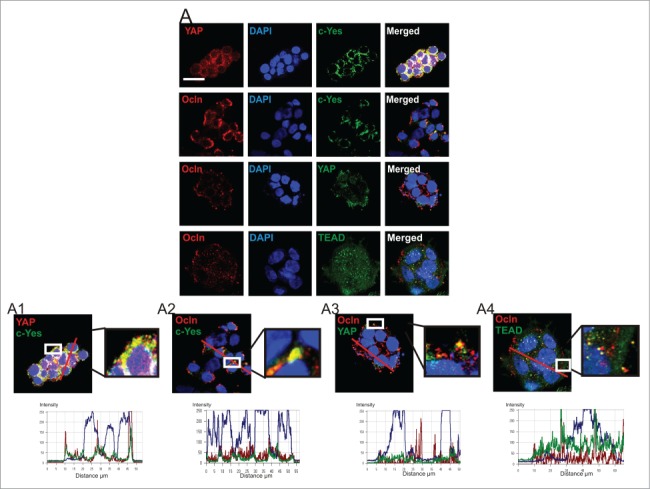
**Distribution and co-localization of c-Yes, Ocln, YAP, and TEAD in HPAFII cells** ***in vitro***. (**A**) Immunofluorescence distribution and confocal co-localization of YAP and c-Yes, Ocln and c-Yes, Ocln and YAP, and Ocln and TEAD. Higher magnifications of co-localizations are presented (**A1–4**). Graphs showing overlapping fluorescence intensity are presented below associated micrographs with red line denoting path of analysis; scale bar, 20 μm.

HPAFII cells are a human pancreatic cancer cell line that demonstrates the capacity to establish functional TJs and differentiate *in vitro* to form polarized monolayers on permeable supports (Kim et al., 1989). We first characterized the cellular distribution for proteins of interest in HPAFII cells grown at low cell density on plastic where nascent lateral cell-cell contacts had begun to be established. Immunofluorescence demonstrated YAP was extensively co-localized with c-Yes. This co-localization occurred primarily in the cytoplasm; some YAP, but very little c-Yes was observed in the nucleus (**Fig. 1A1**). Ocln/c-Yes co-localizations were observed primarily at cell-cell contacts but these appeared to occur to a lesser extent than c-Yes/YAP co-localizations and showed similarities to the Ocln/YAP distribution: mostly cytoplasmic co-localizations (**Fig. 1A2**). Co-localization of Ocln with YAP was less striking relative to Ocln/c-Yes and c-Yes/YAP interactions (**Fig. 1A3**). Further, c-Yes/YAP co-localizations appeared to be primarily in the cytoplasm while Ocln/YAP co-localizations were observed more frequently at leading edge surfaces of these small cell colonies. TEAD/Ocln co-localizations were predominately in the cytoplasm of HPAFII cells, with limited interactions at the cell surface (**Fig. 1A4**). Higher magnification analysis showed TEAD/Ocln co-localizations to be incomplete in their overlap (**Fig. 1A4**), unlike the more complete overlay observed for c-Yes/YAP, c-Yes/Ocln and YAP/Ocln co-localizations (**Fig. 1A1–A3**).

**Figure 2.**
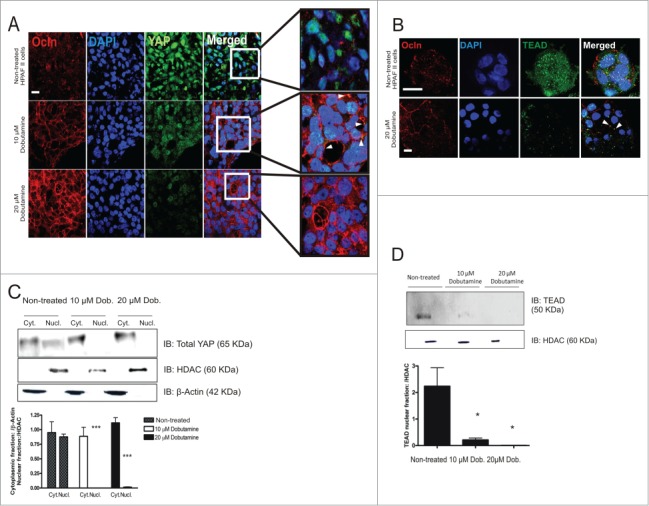
Dobutamine induces YAP and TEAD translocation from the nucleus and stabilization of Ocln at the cell membrane of HPAFII cells. (**A**) Confocal immunofluorescence microscopy showing YAP and Ocln distribution in HPAFII cells. Cells treated with 10 or 20 μM dobutamine for 24 h were similarly analyzed for YAP and Ocln cell distribution. Scale bar, 20 μm. (**B**) Confocal immunofluorescence microscopy showing TEAD and Ocln distribution in HPAFII cells with control buffer addition vs. 20 μM dobutamine for 24 h; scale bar, 20 μm. (**C**) Immunoblot analysis of total YAP in nuclear and cytoplasmic fractions of HPAF II cells as a consequence of 10 or 20 μM dobutamine (Dob) exposure. HDAC (histone deacetylase) and β-actin were used as loading controls for nuclear and cytoplasmic fractions, respectively. Quantification of YAP expression values are the mean of 3 independent experiments ± SEM; ***P < 0.001. (**D**) Immunoblot analysis of TEAD in the nuclear fractions of HPAF II cells as a consequence of 10 or 20 μM dobutamine exposure. Quantification of TEAD expression values are mean of 3 independent experiments ± SEM; *P < 0.05.

HPAFII cells grown at high cell density on plastic and treated with dobutamine demonstrated a dose-dependent increase in Ocln immunofluorescence localized to the cell surface and decrease in overall and nuclear YAP content relative to controls (**Figs. 2A,C**). Dobutamine is a β-adrenergic receptor agonist that induces YAP translocation from the nucleus to the cytoplasm through a mechanism independent of Hpo signaling (Bao et al., 2011). Based upon previous studies, these changes in YAP presumably occurred through its redistribution to the cytoplasm where it is phosphorylated and degraded as a consequence of dobutamine treatment (Chan et al., 2011). Similarly, dobutamine treatment of HPAFII cells resulted in a dramatic reduction in nuclear TEAD content with the remaining TEAD being notable for its apparent co-localizations with Ocln at the plasma membrane (**Fig. 2B, D**).

### Dobutamine-induced redistribution of Ocln has functional consequences

**Figure 3.**
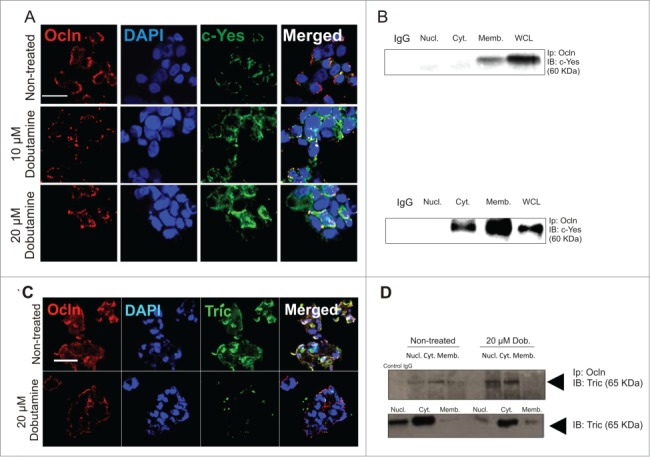
Redistribution of Ocln and c-Yes in HPAFII cells and restriction of tricellulin to tri-cellular contacts following dobutamine treatment. (**A**) Confocal microscopy showing c-Yes and Ocln distribution in HPAFII cells. Cells were treated with 10 or 20 μM dobutamine for 24 h period prior to c-Yes and Ocln cell distribution analysis. Scale bar, 20 μm. (**B**) Immunoprecipitation of Ocln followed by an immunoblot analysis for c-Yes in nuclear, cytoplasmic, and membrane fractions of non-treated and 20 μM dobutamine treated HPAFII cells. (**C**) Confocal images showing distribution of Ocln and tricellulin (Tric) in HPAFII cells treatment with 20 μM dobutamine for 24 h. Scale bar, 20 μm. (**D**) Immunoprecipitation of Ocln followed by an immunoblot analysis for Tric in nuclear, cytoplasmic, and membrane fractions of HPAFII cells following 20 μM dobutamine exposure. *Abbreviations: Nucl (Nuclear fraction); Cyt (Cytosolic fraction); Memb (Membrane fraction)*.

Dobutamine treatment of HPAFII cells grown at low cell densities resulted in a dose-dependent shift in Ocln immunofluorescence from the cytoplasm to the cell surface (**Fig. 2**). Concomitant with this shift in Ocln localization, there was an increase in co-localizations involving c-Yes and Ocln that was associated predominantly with the membrane fraction of these cells (**Fig. 3A, B**). These results are interesting in light of previous findings showing that c-Yes is associated with Ocln at assembling TJ structures and is dissociated from Ocln at times when TJ structures are disassembling (Chen et al., 2002b). Thus, suppression of YAP translocation to the nucleus by dobutamine treatment is associated with increased levels of c-Yes/Ocln co-localization in polarizing HPAFII cells.

Besides Ocln, 2 other MARVEL family proteins localize to TJ structures: MARVELD2 (tricellulin: Tric) and MarvelD3 (Md3) (Mariano et al., 2011). While Ocln and MarvelD3 co-localize at bicellular contacts (Kojima et al., 2011), Tric is normally restricted to tricellular contacts (Ikenouchi et al., 2005). Similar to Ocln, down-regulation of Md3 is associated with EMT in pancreatic cancer cells; Tric knockdown studies suggest it to have a role in the size-selective properties of TJ barriers rather than EMT events (Mariano et al., 2011). Others have previously described how functional Ocln expression can drive Tric from bi-cellular to tri-cellular contact sites in developing epithelial barriers (Ikenouchi et al., 2005). We examined the impact of dobutamine on Tric organization in HPAFII cells forming cell-cell contacts at low cell density (**Fig. 3C**). Untreated HPAFII cells demonstrated Ocln/Tric co-localizations to be present at both bi-cellular contacts and at the external facing cell surfaces of cell colonies. Dobutamine treatment resulted in the exclusion of Tric from bi-cellular contacts that were subsequently dominated by Ocln. Interestingly, Ocln/Tric co-localization remained at externally directed surfaces of cells where no bi-cellular contacts could be established. Immunoprecipitation studies verified the shift of Tric to the membrane following dobutamine treatment but with an increase in its associations with Ocln (**Fig. 3D**). Together, the findings of c-Yes co-localization and Tric restriction to tri-cellular contact sites suggest that Ocln expression and redistribution to the plasma membrane induced by dobutamine treatment has functional consequences.

### Dobutamine accelerates and enhances epithelial cell apical-basal polarization

**Figure 4.**
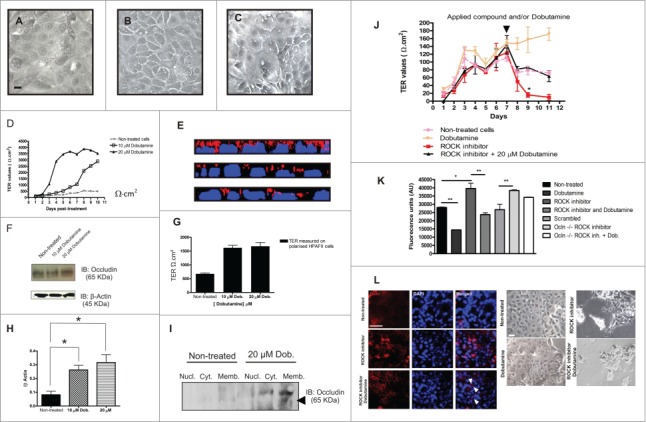
**Dobutamine treatment of polarized cell sheets of HPAFII cells**. Bright field light micrograph of confluent HPAFII cells grown on plastic without treatment (**A**), or after 24 h exposure to 10 μM dobutamine (**B**) or 20 μM dobutamine (**C**); scale bar, 50 μm. (**D**) TER values of HPAFII cell sheets cultured on permeable supports in the absence or presence of dobutamine. (**E**) Z-stacks of non-treated polarized HPAF II cells (top panel) or HPAFII monolayers exposed to 10 μM or 20 μM of dobutamine (middle and bottom panel respectively); red (Ocln), blue (cell nuclei). (**F**) Immunoblot for total Ocln in HPAFII cell monolayers at day 10. (**G**) TER values acquired on day 10 – note this is a separate study from data shown in (D). (**H**) Quantification of total Ocln in HPAFII cells at day 10 culture comparing non-treated with cells exposed to dobutamine; values are mean of 3 independent experiments ± SEM; *P < 0.05. (**I**) Immunoblot for Ocln in HPAFII cells present in isolated fractions before and after treatment with dobutamine. (**J**) TER measurements in HPAFII confluent monolayers seeded in Transwell® filters treated on day 7 with 20 μM Y-27632 or 20 μM dobutamine, either alone or in combination; values are mean of 3 independent experiments ± SEM; *P < 0.05; (**K**) Paracellular permeability of HPAFII cells cultured on trans-well filters, using 4 KDa fluorescently labeled FITC-Dextran - 20 μM of Y-27632 or 20 μM of dobutamine, either alone or in combination, was added to the apical chamber on day 11; afterwards fluorescence levels were measured in the receiver outer chamber; Values are mean of 3 independent experiments ± SEM, *P < 0.5,**P < 0.01; (L-left hand side) Confocal microscopy images of HPAFII cells seeded on trans-well filters, after being exposed to 20 μM Y-27632 either alone or in combination with 20μM dobutamine compared to non-treated cells; Scale bar, 20 μm; (L-right hand side) Bright field images of HPAFII cells seeded on 6 well plates after being treated with 20 μM Y-27632 or 20 μM dobutamine, either alone or in combination, compared to non-treated cells; scale bar, 50 μm. *Abbreviations: Nucl (Nuclear fraction); Cyt (Cytosolic fraction); Memb (Membrane fraction)*.

Confluent cultures of HPAFII cells grown on plastic demonstrated changes in cell-cell contacts without striking differences in cell number, size, or shape when treated with increasing concentrations of dobutamine (**Fig. 4A–C**). HPAFII cells grown as confluent monolayers on permeable supports can complete apical-basal polarization, resulting in polarized sheets with trans-epithelial electrical resistance (TER) values of ˜300–500 Ω·cm^2^ by day 8 of culture (**Fig. 4D**). Addition of dobutamine accelerated and enhanced the TER values of HPAFII cell sheets in a dose-dependent manner, with TER values increasing to as high as ˜4,000 Ω·cm^2^ (**Fig. 4D**) that typically plateaued at ˜1,700 Ω·cm^2^ (**Fig. 4G**). Dobutamine treatment altered the localization of Ocln that was consistent with its consolidation at TJ structures in HPAFII cell monolayers (**Fig. 4E**) that coincided with an increase in total Ocln levels (**Fig. 4F, H**) and increased TER (**Fig. 4G**). Further, immunoblot analysis verified that dobutamine treatment increased the apparent molecular weight of Ocln at the membrane (**Fig. 4I**), consistent with an increased phosphorylation status (Dorfel and Huber, 2012).

PKC-ζ and Rho kinase have both been demonstrated to play critical roles in the organization and stabilization of functional TJs (Dodane and Kachar, 1996; Etienne-Manneville and Hall, 2002; Stuart and Nigam, 1995; Suzuki et al., 2001), with PKC-ζ directly interacting with Ocln (Nusrat et al., 2000). We tested the potential involvement of PKC-ζ and ROCK in processes involving dobutamine enhancement of TJ function through pharmacological suppression using the protein kinase C zeta pseudosubstrate (PKC-ζ-PS) peptide inhibitor (Lim et al., 2008) and the p160ROCK inhibitor Y-27632 (Ishizaki et al., 2000; Matthews et al., 2006). Incubation of HPAFII monolayers with PKC-ζ-PS ablated TER and treatment with dobutamine failed to have any effect on this outcome (**Fig. S.1**). Treatment with Y-27632 also ablated TER, but in this case treatment with dobutamine could counteract the actions of Y-27632 (**Fig. 4J, K, L**). These data are consistent with the previous roles of PKC-ζ activity being critical for TJ structure/function through its actions on Ocln function while Rho regulation of TJ organization is not as directly aligned with Ocln function at TJs (Ishizaki et al., 2000; Matthews et al., 2006; Nusrat et al., 2000).

### Assessment of Ocln with Hpo pathway elements in cells incapable of polarization

**Figure 5.**
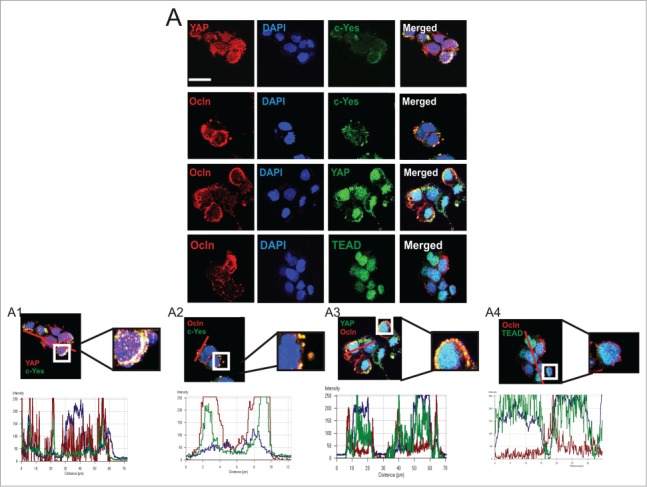
**Distribution and Co-localization of c-Yes, Ocln, YAP, and TEAD in AsPc1 cells** ***in vitro***. (**A**) Immunofluorescent distribution and confocal co-localization of YAP and c-Yes, Ocln and c-Yes, Ocln and YAP, and Ocln and TEAD. Higher magnifications of co-localizations are presented (**A1–4**). Graphs showing overlapping fluorescence intensity are presented below associated to the micrograph with red line denoting path of analysis; scale bar, 20 μm.

AsPc1 is a cell line established from a human pancreatic ductal adenocarcinoma (PDAC); it is known for its aggressive growth characteristics and inability to form functional TJ structures (Tan and Chu, 1985). When grown at low cell density as previously described for HPAFII cells (**Fig. 1**), AsPc1 cells expressed Ocln at levels comparable to or even greater than those observed in HPAFII cells with some immunofluorescence being associated with the plasma membrane (**Fig. 5A**). YAP/c-Yes co-localization occurred mostly in the cytoplasm (**Fig. 5A1**). Ocln/c-Yes co-localized predominantly at the membrane similar to that observed in HPAFII cells (**Fig. 5A2**). The striking differences between HPAFII and AsPc1 cells were primarily related to YAP and TEAD distribution with a much greater relative expression in the nucleus for both in AsPc1 cells (**Fig. 5A3 and A4**). Ocln/YAP co-localization was extensive at and adjacent to the plasma membrane (**Fig. 5A3** and **Fig.S.2**), different from that observed for HPAFII cells. TEAD/Ocln co-localization was almost non-existent (**Fig. 5A4**) and failed to show cytoplasmic co-localizations as observed in HPAFII cells.

We next examined the actions of dobutamine treatment on AsPc1 cells; a dose-dependent loss of nuclear YAP in AsPc1 cells was associated with an increase in Ocln expression (**Fig. S2**). There was also a loss of YAP/Ocln co-localizations at or near the plasma membrane and the distribution of Ocln shifted from being more pronounced at external faces of cells to cell-cell contact locations (**Fig. S2**). Dobutamine treatment of AsPc1 cells produced an increase in the extent of c-Yes/Ocln co-localization observed at or near the plasma membrane, similar to HPAFII cells (**Fig. S3**). Changes in the cellular distribution of TEAD in AsPc1 cells induced by dobutamine treatment mirrored those observed for YAP (**Fig. S.4**). It should be noted that we failed to observe such striking changes in YAP/Ocln or TEAD/Ocln co-localizations in HPAFII cells following dobutamine treatment (**Fig. 2**). We next examined the relative cellular distributions for YAP and Ocln associations by immunoprecipitating YAP followed by immunoblotting for Ocln (**Fig. S5**). These studies showed that showed that Ocln in HPAFII cells was more extensively membrane-associated than in AsPc1 cells but that less of the Ocln in HPAFII cells was associated with YAP at the membrane compared to AsPc1 cells. Note that most of the Ocln-antibody reactive materials were of lower molecular weight than full-length Ocln, suggesting the presence of catabolic fragments.

### Actions of dobutamine on HPAFII and AsPc1 cell proliferation

**Figure 6.**
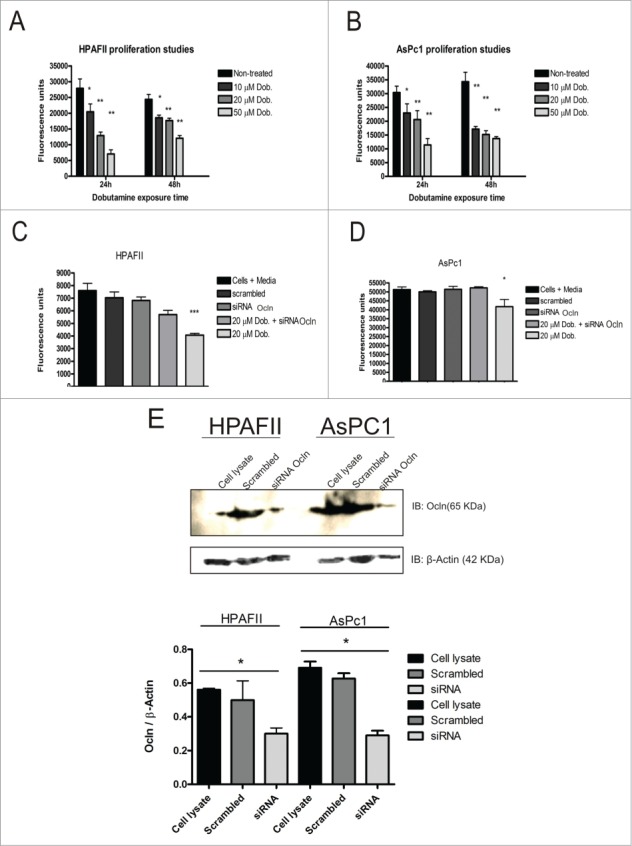
For figure legend, see page 9. **Figure 6 (See previous page).** **Dobutamine suppresses proliferation through Ocln**. Cell proliferation of HPAF II cells (**A**) and AsPc1 cells (**B**) exposed to 10, 20 and 50 μM Dobutamine over 24 h and 48 h; values are expressed as mean ± SEM from 3 independent experiments; *P < 0.05; **P < 0.01 compared to non-treated cells. HPAFII (**C**) and AsPc1 (**D**) cells treated with siRNA targeting Ocln or a scrambled, control siRNA at the same time as dobutamine was added to initiate the time course of the study. Cell proliferation was determined using a live cell fluorescent label. Values are mean of 3 independent experiments compared to siRNA treated levels ± SEM; *P < 0.05; ***P < 0.001. (**E**) Immunoblot showing Ocln knockdown and quantification of protein expression.

The transcriptional co-activator complex YAP/TEAD is known to affect cell proliferation rates (Chen et al., 2010). Treatment with dobutamine inhibited the proliferation of both HPAFII and AsPc1 in a dose-dependent manner after 24 h and 48 h of exposure (**Fig. 6A, B**). Transient knockdown of Ocln expression using siRNA did not significantly alter the proliferation rate of HPAFII cells, but it did significantly impede the ability of dobutamine to suppress HPAFII cell proliferation (**Fig. 6C**). Similar, but less striking findings were observed for AsPc1 cells (**Fig. 6D**). It should be noted that the impact of siRNA or control (scrambled) siRNA added at the initiation of these experiments would have taken several hours to affect Ocln expression levels, reducing the potential impact of these treatments over the time course of this study (**Fig. 6E**). Despite this caveat, these studies suggest that dobutamine reduced proliferation rates in these 2 pancreatic cancer cell lines, possibly through a mechanism involving Ocln.

### Expression pattern of c-Yes, Ocln, YAP and TEAD in human pancreatic biopsies

**Figure 7.**
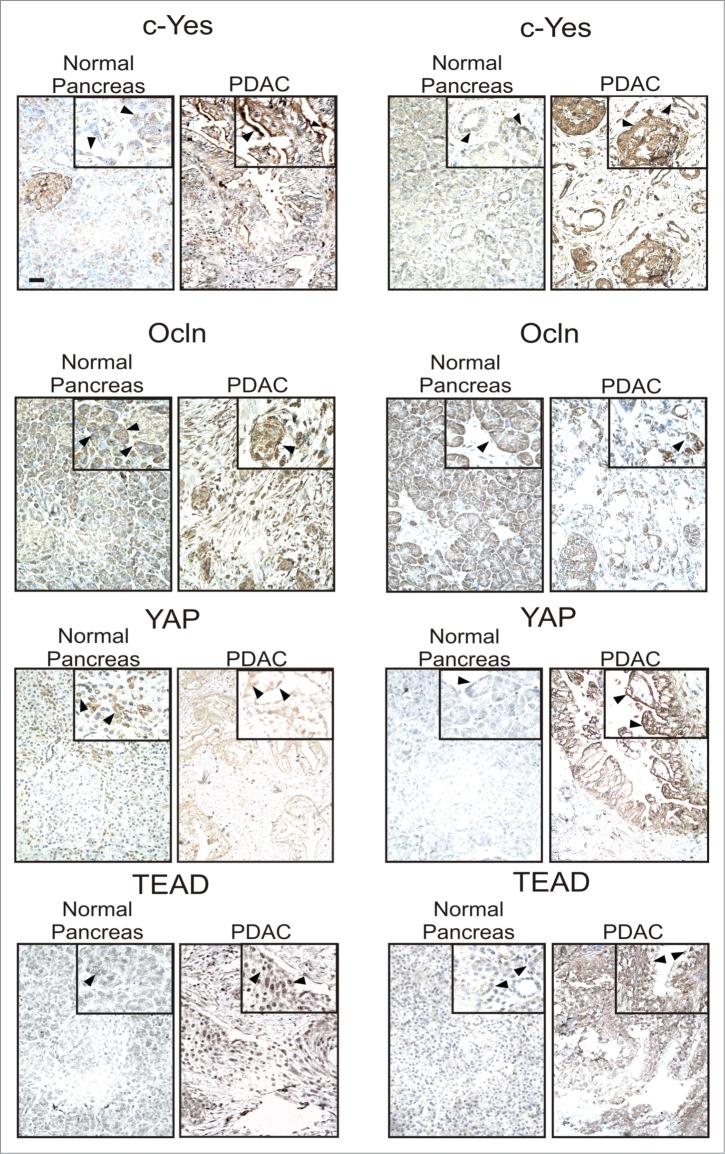
**Immunostaining of normal and cancerous pancreatic tissue biopsies**. Individual biopsies, obtained from donation (normal) or surgical biopsy (PDAC), were fixed, embedded, sectioned, mounted on slides, and stained with specific antibodies following an antigen-retrieval protocol. Each picture represents one image field per patient sample biopsy. Antibody labeling was detected using a peroxidase reaction prior to counter-staining with Meyer´s Haematoxylin. Scale bar, 50 μm.

Our *in vitro* data with 2 different human pancreatic cancer cell lines has suggested a potential role for Ocln to regulate cell proliferation through the Hpo signaling cascade. Ocln co-localization with YAP and TEAD suggested a role in the establishment/stabilization/organization of cell-cell contacts associated with TJs. Comparison of epithelial cells capable of undergoing apical-basal polarization (HPAFII) with those that cannot undergo this differentiation (AsPc1) demonstrated that nuclear localization of the YAP/TEAD complex was decreased when Ocln expression was increased by dobutamine treatment. To better understand how these observations might apply to clinical status, we examined human pancreatic biopsies from 4 different patients to assess the expression profile and localization of c-Yes, Ocln, YAP and TEAD in cancerous and non-cancerous conditions (**Fig. 7**, **Fig. S6 and S7**). In normal (non-cancerous, non-inflamed) pancreas we noted that c-Yes was distributed in the cytoplasm and in small amounts at the apical plasma membrane of duct cells. A moderate to strong nuclear c-Yes staining was observed in 2 pancreatic cancer tissue biopsies and a strong signal was also detected at the apical surface of cells lining duct-like structures, which agrees with the distribution of this non-receptor tyrosine kinase in AsPc1 cells.

Ocln expression in normal pancreas samples was very similar to that of c-Yes with localization in the cytoplasm and at the apical plasma membrane of ducts. Ocln expression in PDAC specimens appeared to be substantial but not at the plasma membrane. YAP expression in normal pancreatic epithelial cells was mostly detected in the cytoplasm with minimal nuclear staining observed; stronger YAP staining was detected in biopsies from PDAC patients, which was localized to the cytoplasm and apical surfaces of morphologically altered epithelial cells. TEAD labeling in normal pancreas was restricted to the cytoplasm and very minor amounts of nuclear localization; PDAC samples showed some cytoplasmic staining but also more extensive nuclear localization. Overall, in this limited set of biopsies, we observed c-Yes, Ocln, YAP, and TEAD expression in PDAC that was either altered in its cellular distribution or increased compared to non-cancerous tissue. Ocln, c-Yes, YAP, and TEAD localization in epithelial cells of normal pancreas were more similar to the distribution of these proteins in HPAFII cells while the distribution of these proteins in PDAC biopsies was more consistent with the findings of expression and distribution observed in AsPc1 cells.

## Discussion

The Hpo pathway provides important growth regulation of epithelia cells by using homotypic extracellular contacts made by E-cadherin at AJ structures as a sensor to lateral cell-cell contact status (Gumbiner and Kim, 2014). We hypothesized that epithelia could utilize similar homotypic extracellular contacts at TJ structures as a sensor to signal apical-basal polarity status to suppress proliferation in fully polarized conditions. Our studies using 2 pancreatic cancer cell lines adapted for *in vitro* cell culture provide a potential scenario of how Ocln co-localization with c-Yes, YAP, and TEAD might function as a sensor system for Hpo pathway signaling and build upon the previously established status of c-Yes as a *bona fide* binding partner of YAP and Ocln (Nusrat et al., 2000).

In the case of HPAFII cells, where functional TJs can form, it appears that c-Yes/Ocln co-localize at cell-cell contact regions, consistent with the role of this non-tyrosine kinase to facilitate the formation of functional TJs (Chen et al., 2002b) while c-Yes co-localizes with YAP in the cytoplasm adjacent to these cell-cell contact regions (Sudol, 1994). YAP and TEAD both showed co-localization with Ocln that was restricted to the cell surface and cytoplasm of these cells. Treatment with dobutamine increased Ocln expression and distribution at the plasma membrane, events that coincided with enhanced TJ maturation and decreased YAP and TEAD levels. Dobutamine suppressed cell proliferation rates in an Ocln-dependent fashion that required the actions of PKC-ζ, a kinase that promotes apical-basal polarization and interacts with the C-terminal tail of Ocln (Dodane and Kachar, 1996; Nusrat et al., 2000; Stuart and Nigam, 1995).

In comparison to HPAFII cells, AsPc1 cells fail to form functional TJs and demonstrate distinct co-localization patterns for Ocln with c-Yes, YAP, and TEAD. Most striking, the nuclear levels of YAP and TEAD in AsPc1 cells were increased relative to HPAFII cells. Treatment of AsPc1 cells with dobutamine increased Ocln levels and diminished nuclear YAP and TEAD that coincided with decreased proliferation rates. This suggests that while the sensor function of Ocln might be based upon its localization at TJs, pharmacological intervention with dobutamine can overcome the requirement of increasing Ocln expression levels. Thus, the demonstration that Ocln co-localizes with TEAD and YAP may represent a stoichiometric relationship that sequesters critical Hpo elements to limit their access to the nucleus and their actions on proliferation. Previous studies have suggested that TJs not only establish barrier and fence functions associated with mature epithelium, but also act in cell signaling events (Matter and Balda, 2003). Our studies now highlight the potential for a specific TJ element, Ocln, to participate in a signaling event that suppresses proliferation.

The function of Ocln has been poorly understood since its identification as the first integral membrane protein of the TJ (Furuse et al., 1993), with functional TJ structures forming in the absence of Ocln (Saitou et al., 2000). Our findings support a role for Ocln in regulating bi-cellular TJ structures (Furuse, 2010). Recent studies support the potential for other TAMP family member, Tric and MarvelD3, to replace Ocln absent at bi-cellular contacts (Cording et al., 2013; Raleigh et al., 2011). This compensation does not always, however, provide a complete correction. While OCLN -/- mice demonstrate morphologically and functionally normal TJs and reach adulthood, they exhibit a complex phenotype that includes reduced growth rates, brain calcifications, testicular atrophy, loss of cytoplasmic granules in salivary gland striated duct epithelial cells, and thinning of compact bone (Saitou et al., 2000). Important to this discussion is the fact that OCLN -/- mice also exhibit chronic inflammation and hyperplasia of their gastric epithelium, indicating that Ocln is involved in the balance between epithelial proliferation and differentiation (Furuse, 2009).

Epithelial inflammation is associated with diminished barrier function and the internalization of TJ proteins, such as Ocln, with minimal effects on the cytoplasmic plaque proteins and cytoskeleton associated with TJs (Bruewer et al., 2003). Other studies have shown Ocln to modulate epithelial barrier function (Yu et al., 2005) and expression of Ocln lacking its cytoplasmic domain disrupts apical-basal polarity (Balda et al., 1996). Chronic inflammation and loss of apical-basal polarity are associated with epithelial cell transformation. Such changes, in light of the current observations, are consistent with a potential sensor role for Ocln at TJ structures. This concept is further supported by the demonstration that claudin proteins serve as the essential elements of functional TJs, diffuse slowly in the membrane, and have durable periods of residence at TJs compared to Ocln (Shen et al., 2011), making the more dynamic nature of Ocln membrane residence an appealing aspect for an apical-basal polarity sensor. This combination of rapid dynamics at the plasma membrane and the ability to form homotypic interactions between adjacent cells provides an attractive mechanism for epithelial barriers to assess proliferation *vs*. differentiation programming for the cell.

We observed differences in the co-localization of c-Yes and YAP with Ocln in HPAFII and AsPc1 cells that were consistent with Ocln plasma membrane residence where TJs do or do not form. Importantly, c-Yes, a *bona fide* YAP binding partner is one of the kinases that directly binds to and regulates Ocln function at TJs (Chen and Lu, 2003; Chen et al., 2002b; Nusrat et al., 2000). This is consistent with the observation that epithelial cancers involving dysfunctional Hpo pathway elements have been identified (Pan, 2010) and epigenetic silencing of Ocln promotes tumorigenesis (Osanai et al., 2006). Examination of human normal and PDAC biopsies are consistent for changes previously noted for Ocln and c-Yes in pancreatic cancer (Tan et al., 2004; Kubo et al., 2009). Additionally, YAP and TEAD represent critical components of Hpo signaling, playing a central role in the context of cell proliferation associated with PDAC (Shao et al., 2014). Since YAP does not contain an intrinsic DNA-binding domain but instead regulates target genes by interacting with DNA-binding transcription factors in the nucleus such as TEAD (Yu and Guan, 2013;Vassilev et al., 2001), it is interesting to note that both YAP and TEAD co-localize with Ocln, providing a potential a sensor to relay information relevant to the extent apical-basal polarization through YAP and TEAD to ensure a bias towards polarization *versus* proliferation.

These co-localizations with c-Yes, YAP, and TEAD as well as the actions of dobutamine suggest a mechanism whereby Ocln can function as a sensor at TJ structures. Ocln expression enhanced by dobutamine treatment restricted Tric from bi-cellular contacts and increasing epithelial barrier properties, findings consistent with previous studies examining Ocln function (Ikenouchi et al., 2005; Ikenouchi et al., 2008). Dobutamine enhanced interactions with c-Yes and PKC-ζ, and provided a form of Ocln that could co-localize with YAP and TEAD at the plasma cell membrane and within the cytoplasm. Such a mechanism would limit YAP and TEAD access to the nucleus and lead to the suppression of proliferation that is associated with differentiated epithelia. Knockdown of Ocln levels or inhibition of PKC-ζ led to the release of this suppression, consistent with the idea that functional TJ structures involving Ocln are required for this mechanism to work properly under non-pathological conditions. Overall, our data suggest that despite the fact that the role of Ocln and YAP interaction is not necessarily mediated through a functional TJ complex, Ocln is uniquely positioned at TJ structures to register the extent of a functional barrier for the coordination of apical-basal polarity with cell density through co-localization with Hpo pathway elements (YAP and TEAD).

## Materials and Methods

### Cell culture, TER measurements, paracellular permeability

AsPc1 and HPAF II cell lines were obtained from ATCC, were passaged 2 months after receipt and were authenticated and characterized through Short Tandem Repeat (STR) profiling. Cells were maintained at 37°C in a humidified atmosphere containing 5% CO_2_. AsPc1 cells were maintained in RPMI 1640 media (Fisher^©^ / cat. number - 10418243) and HPAFII in MEM (Fisher^©^ / cat. number - 11514426), both supplemented with 10% fetal bovine serum FBS (Invitrogen / cat. number - 10500064) penicillin (100 U/mL) and streptomycin (100 mg/mL) (Sigma^©^ / cat.number - P4333). Cells were sub-cultured twice weekly at a 1:2 split ratio. For apical-basal polarity studies, 70,000 HPAFII cells per well (between passage 10 and 12) were seeded onto 0.4-μm pore size, 12 mm Transwell™ filters (Fisher^©^ / cat. number - CLS3460); TER values were measured by fixed paddle electrodes (World Precision Instruments^©^, UK). For paracellular studies, TER measurements were performed on a daily basis and on day 7, cells were treated with 20 μM ROCK inhibitor Y-27632 dihydrochloride (Sigma^©^ / cat- number - Y0503) or 50 μM PKC-ζ pseudosubstrate (PKC ζ-PS) (Fisher^©^ / cat. number - 10717873) either alone (Cerruti et al., 2013) or in combination with 20 μM Dobutamine (Sigma^©^ / cat. number - D0676). Culture media was replaced by fresh MEM without phenol red (Fisher^©^ / cat. number - 51200–038) and cultures were then left to equilibrate during at least 30 minutes. FITC Dextran (Sigma^©^ / cat. number – 46944) was added to the inner chamber to a final concentration of 1 mg/mL. Cultures were then incubated at 37°C for 4 h. Filters were then removed and the diffused tracer measured by the FLUOstar OPTIMA (FITC-Dextran: Excitation: 485 nm and emission: 544 nm). Each sample was analyzed in triplicates from independent experiments.

### Protein extraction and subcellular fractionation

To collect cytoplasm fractions, growth media was removed and cells were washed twice in ice-cold PBS prior to the addition of RIPA buffer (Fisher^©^ / cat. number – 10017003). After incubation on ice for 5 minutes, cells were collected using a cell lifter and the lysate clarified by centrifugation (8000 xg, 10 min, 4°C). Preparation of protein concentrations was determined using a NanoDrop 2000 instrument (Thermo Scientific). For membrane isolation, cells were washed with PBS containing Ca^2+^ and Mg^2+^ and lifted into 50 mM Tris/HCl, pH 7.4 with protease (Fisher^©^ / cat. number – 12841640) and phosphatase inhibitors (Fisher^©^ / cat. number – 12851650) while on ice. Collected cells were disrupted by freeze-thaw cycles prior to centrifugation (1000 xg, 10 min, 4°C). After centrifugation, nuclear fractions were collected and suspended in lysis buffer. Membrane pellets were isolated from clarified supernatants by ultracentrifugation at 100,000 xg, for 1 h at 4°C (Beckman). Pellets were washed in this way twice in PBS containing Ca^2+^ and Mg^2+^ and then suspended in lysis buffer containing 0.1% Triton-X-100 (Sigma^©^ / cat. number - T8787). Each experiment was repeated 3 times.

### Immunoprecipitation and immunoblotting

Cells were lysed in RIPA buffer for 10 min at 4°C and the preparation centrifuged (10,000 xg, 10 min, 4°C). Lysates containing 500 μg of protein were incubated with a pull-down antibody overnight at 4°C. A/G beads™ (Santa Cruz Biotech / cat. number - sc-2003) were added and the mixture was incubated with rotation for 2 h at 4°C. Beads were washed 3 times in RIPA buffer by centrifugation at 4000 xg, 1 min, RT. 20–35 μg of the protein extract was separated by 12% SDS PAGE (100–150 V, 45 min) and transferred (XCellTM Blot module; Invitrogen) onto a PVDF membrane (Thermo Scientific / cat. number – 10617354) at 30 V for 1.5 h. The PVDF membrane was then rinsed briefly in ddH20 and blocked in 5% milk dissolved in TBS-T (10 mM Tris- HCl, pH 7.5, 150 mM NaCl and 0.1% Tween 20) for 1 h at RT. Blots were then incubated overnight at 4°C in TBS-T containing 5% BSA (Sigma^©^ / cat. number - A7906) and a primary antibody at a 1:500 dilution: Ocln polyclonal rabbit (Invitrogen / cat. number - 33–1500), total YAP rabbit monoclonal (Santa Cruz / cat. number - sc-15407), TEAD mouse monoclonal (Cell Signaling / cat. number - 8526), or c-Yes mouse monoclonal (Santa Cruz / cat. number - sc-8403). After three washes in TBS-T at RT, appropriate secondary-horseradish peroxidase (HRP) antibodies (Rabbit: GE Healthcare / cat. number - NA934; Mouse: GE Healthcare / cat. number: NXA931) were applied at a dilution of 1:1000 for 1h at RT in TBS T containing 1% milk. Blots were washed in TBS-T with HRP activity being detected by ECL (GE Healthcare^©^ / cat. Number - RPN2232).

### Immunohistochemistry

Human pancreatic biopsy tissue sections on glass slides were de-waxed in Histoclear (Fisher^©^ / HIS- -010–010S) for 10 min, rehydrated in 10 min steps through graded ethanol (100%, 90%, 70% and 50%), and then washed in PBS with gentle shaking for 5 min. Heat induced antigen retrieval was performed using hot 10 mM of Citrate buffer pH 6.0 in a pressure cooker (Morphy Richards) for 15 min followed by a second heating for 5 min at a lower temperature (Shi et al., 1993). Tissue sections were blocked for 30 min at RT with serum blocking reagent and incubated in either a primary antibody or control antibody (Rabbit - DA1E, cat. number - 3900 ; Mouse - G3A1, cat. number – 5415; Cell Signaling). Antibodies were used at 1:100 dilution in PBS with 0.1% BSA and incubated overnight at 4°C in a humid chamber. Slides were then washed in PBS, exposed to a biotin-labeled secondary antibody for 30 min at RT, washed in PBS again, and treated with 30% (w/w) H_2_O_2_ to quench the endogenous peroxidase activity (Veitch, 2004). After being washed in PBS, slides were incubated at RT in avidin:biotin enzyme complex (Vecstatin Elite ABC kit, Vector laboratories, cat. number - PK-6101) for 30 min, washed again and incubated with 3,3'–diaminobenzidine (DAB) (Vector Laboratories / cat. number - SK-4100) according to the manufacturer's instructions. Tissue sections were co-stained with Meyer´s Haematoxylin (Sigma^©^ / cat. number - H9627) for 2 min, rinsed with water, mounted in DePeX glue (Fisher^©^ / cat. number - 10050080) and allowed to dry overnight. Images were captured at 20x magnification using a DMI 3000B Microscope (Leica Microsystems) and managed using Adobe Photoshop, version 3.0 software (Adobe Systems, Mountain View, CA).

### Immunofluorescence and confocal microscopy

Cells were grown on coverslips for 48 h, washed 3 times with 10 mM HEPES buffer containing 0.9% CaCl2, and then fixed with 4% PFA for 20 min. Cells were simultaneously blocked and permeabilized overnight at 4°C in PBS containing 2% BSA and 0.5% Triton X-100. Cells were then washed with 10 mM HEPES buffer containing 0.9% CaCl_2_ and incubated for 2 h at 4°C with a primary antibody diluted 1:100 in PBS and then a secondary antibody diluted 1:400 in PBS for 30 min. DAPI (Sigma^©^ / cat. number – 32670) was applied for 20 min, and cells were mounted on mowiol and examined using Zeiss Confocal Laser Scanning Microscope using 3 laser wavelengths: Argon 488 nm, HeNe 543 nm and HeNe 633 nm. Images were captured using the 63x oil immersion objective. Results shown are representative of at least 3 independent experiments.

### Cell proliferation assay

Cells were plated in white clear bottom 96-well plates, and allowed to attach overnight. Test compounds were added in optimal growth media and incubated for 24 h. For cell proliferation assays, treatment media was removed and 100 μL of 1x dye binding solution of CyQuant (Fisher^©^ / cat. number – 10709253) was added. After a 30 min incubation, fluorescent intensity was measured at 22°C using a microplate reader (FLUOstar Omega; BMG Labtech) at 485/530nm excitation/emission wavelengths. Each sample was analyzed in triplicates from independent experiments.

### Ocln Knockdown

We used a pool of 4 individual siRNA (Accell Human OCLN, siRNA-SMARTpool, Fisher^©^ / cat. number - E-187897–00–0005) targeting different sequences in the Ocln gene (details of siRNA used are shown in **Table 1**). For proliferation assays HPAFII and AsPc1 (passage 12) cells were grown on a 96 well plate approximately 70% confluent. A transfection mixture of 1 μL of HiPerfect, 0.02 μL of siRNA (20 μM stock) or 0.6 μL of scrambled siRNA (Fisher^©^ / cat. number – 11841984) were added to 7.5 μL of serum- and antibiotic-free media and rocked for 15 min at RT. 172 μL of fresh media with and without Dobutamine (RPMI or MEM supplemented with 10% FBS, penicillin (100 U/mL) and streptomycin (100 mg/mL) was added to each well; the transfection mix was added in a drop-wise fashion and then cells were incubated at 37°C in 5% CO_2_ for 24 h. Cells were treated in parallel with a scrambled control siRNA. After removal of treatment media, a cell proliferation assay, previously described, was carried out. For protein levels, cells were harvested in RIPA buffer and run on a gel for western blot analysis.

**Table 1.** Sequences of siRNA used to knockdown human Ocln were prepared as SMARTpool and used for transfections

Species	Target	Sequence
Human	Ocln	GCAUUAACUAGCUGGAUGU
Human		CCCGUUUGGAUAAAGAAUU
Human		GUCAGAGGUUUAGAUUAGA
Human		CUCUUCAACUGUUAGAUUC

### Pancreatic tissues and ethics statement

Tissue samples were collected from patients following pancreatic resection for PDAC. Normal pancreatic tissue samples were obtained through an organ donor procurement program whenever there was no suitable recipient for pancreas transplantation. Tissues were fixed in 5% paraformaldehyde solution for 12–24 h and then embedded in paraffin for histological analysis (Erkan et al., 2005). All patients were informed, written consent was obtained, and human protocols were approved by the Ethics Committees of the University of Heidelberg (Germany) and the Technical University of Munich (Germany).

### Statistical analysis

Data are represented as the mean of 3 independent experiments ± SEM. Statistical analysis was performed using GraphPad Prism 5 software. Significant differences between experimental and the control group were determined by one-way ANOVA followed by Dunnet's post hoc test for experiments in which 3 or more groups were compared. A statistically difference was accepted if the p values were less than 0.05. Significance is indicated on the graphs as it follows: * *P* < 0.05, ** *P* < 0.01, *** *P* < 0.001.
