# Supplementary figures and images for: Hippo pathway elements Co-localize with Occludin: A possible sensor system in pancreatic epithelial cells
Source: Tissue Barriers. 2015 May 1;3(3):e1037948. doi: 10.1080/21688370.2015.1037948 (PMC4574897; doi:10.1080/21688370.2015.1037948)

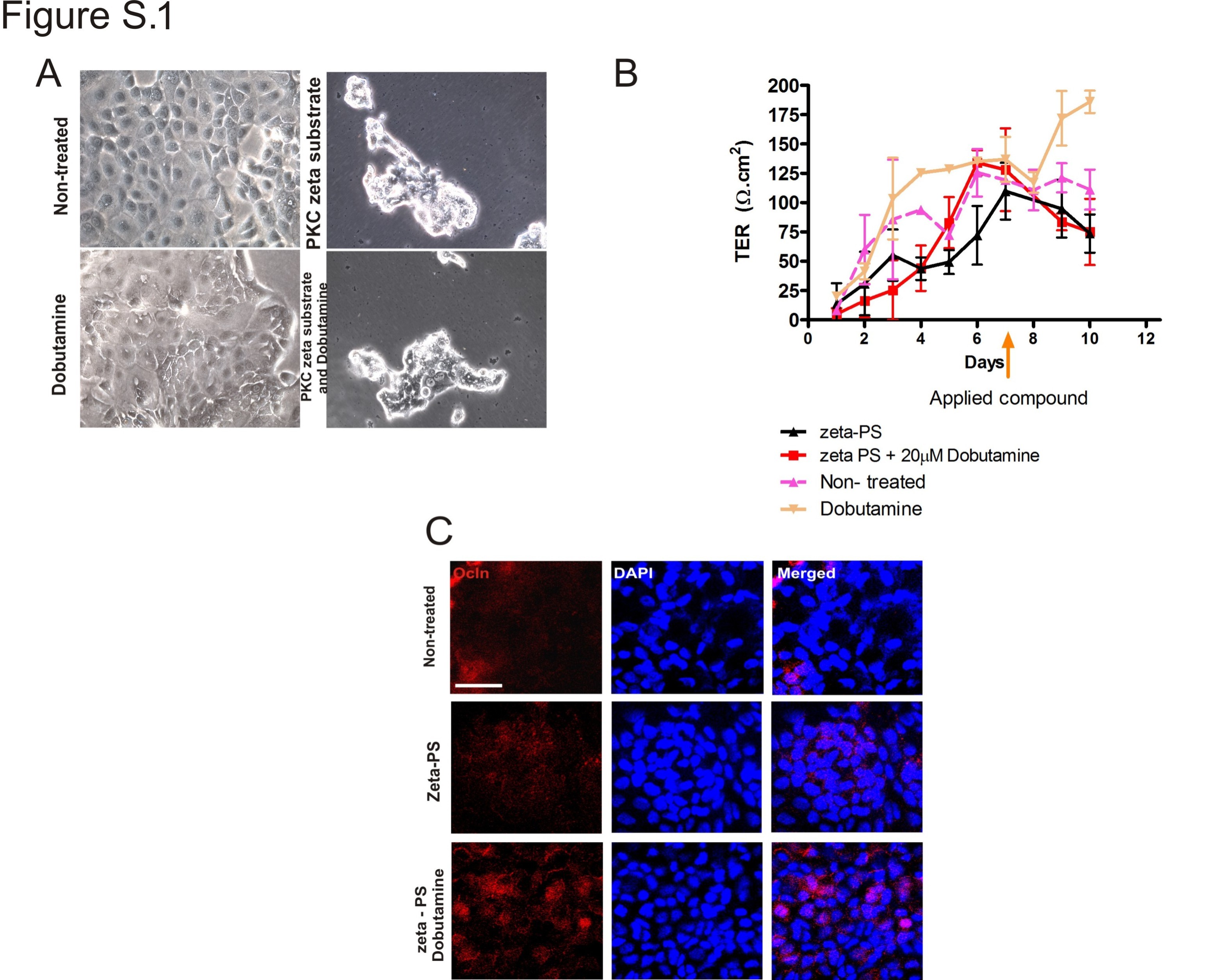

Supplement: Supplemental data for this article can be accessed on the publisher's website. [file ktib-03-03-1037948-s001.zip › Supplemental Material/Supplemental Figure 1.jpg]

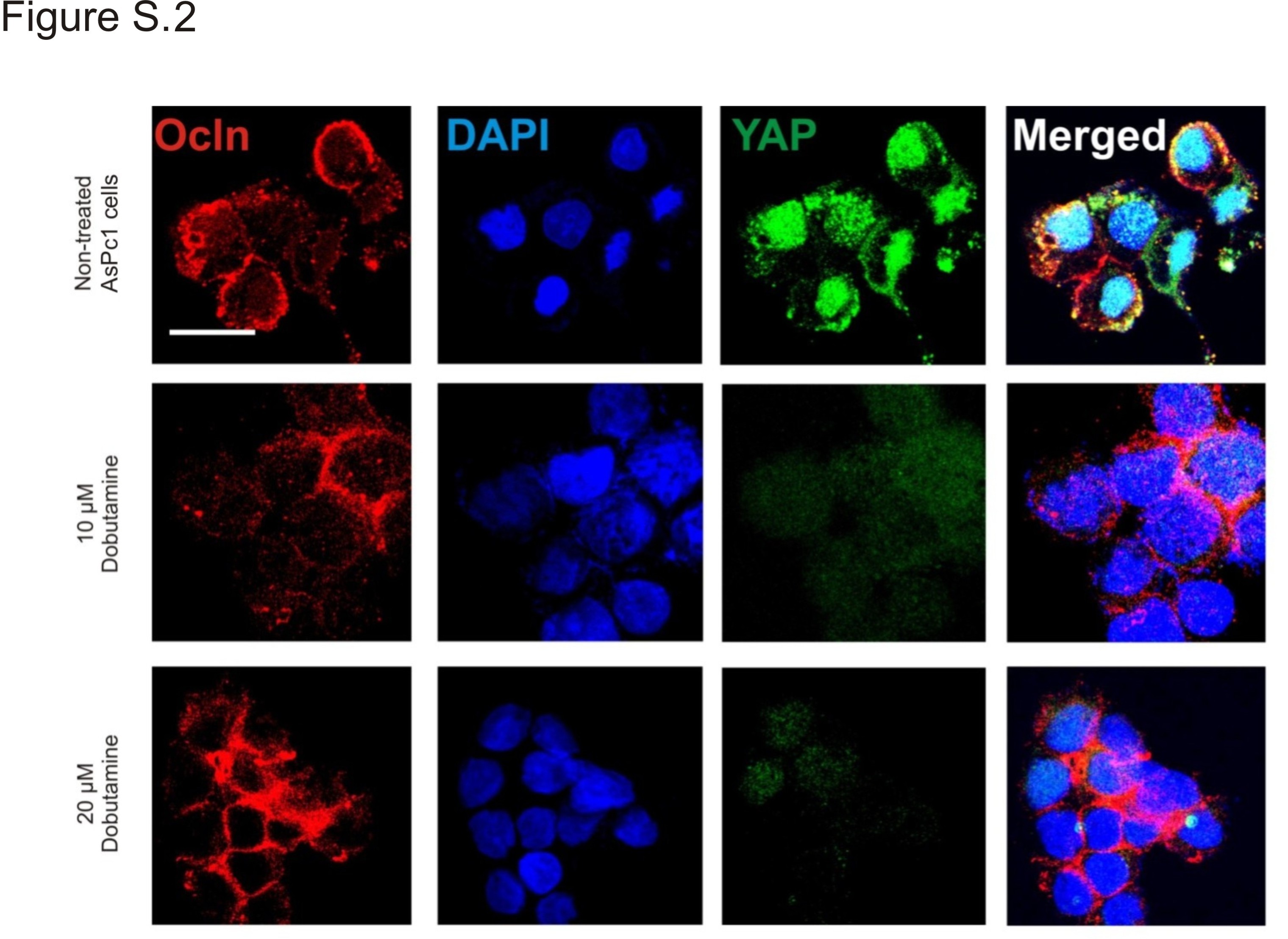

Supplement: Supplemental data for this article can be accessed on the publisher's website. [file ktib-03-03-1037948-s001.zip › Supplemental Material/Supplemental Figure 2.jpg]

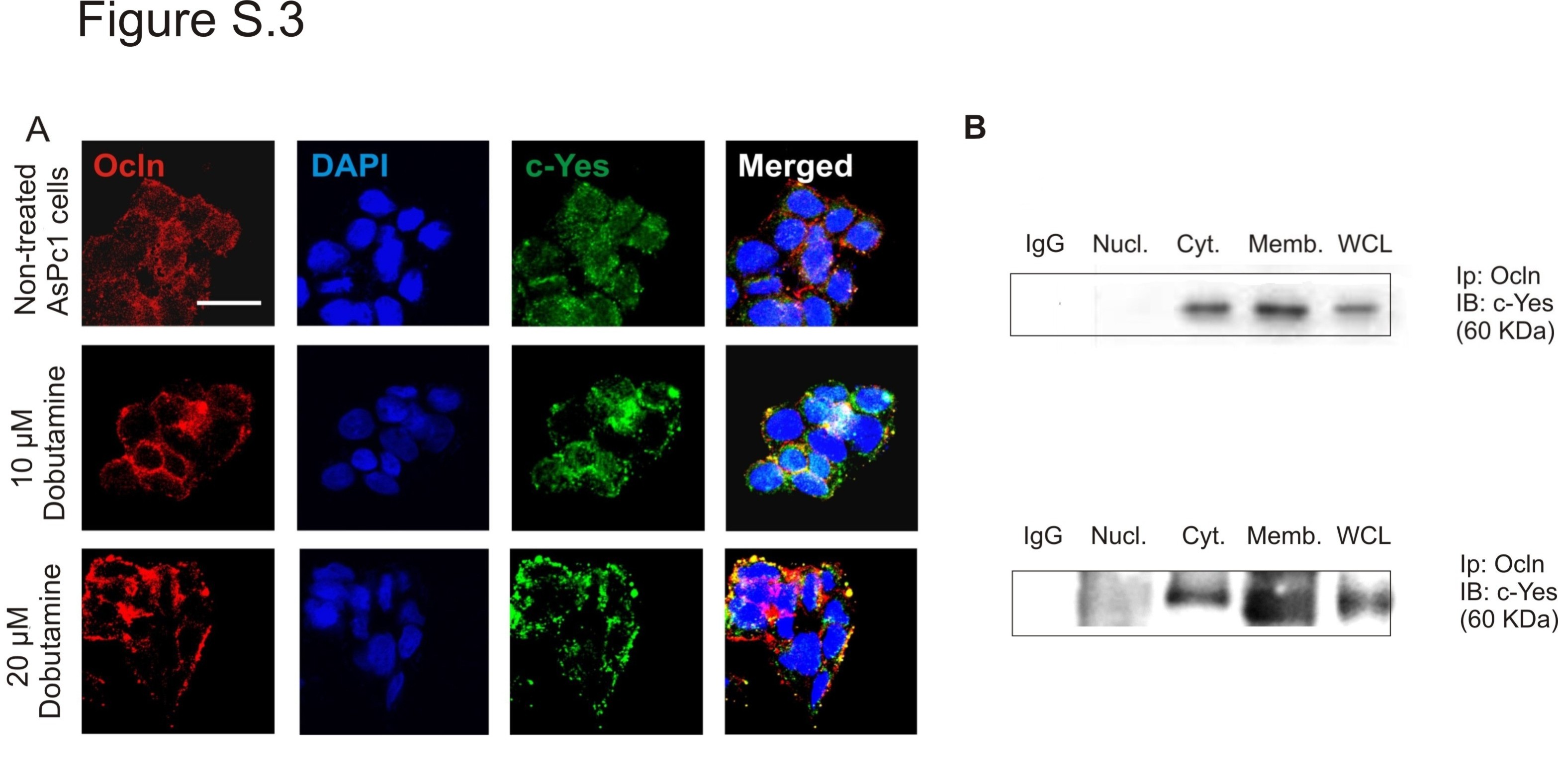

Supplement: Supplemental data for this article can be accessed on the publisher's website. [file ktib-03-03-1037948-s001.zip › Supplemental Material/Supplemental Figure 3.jpg]

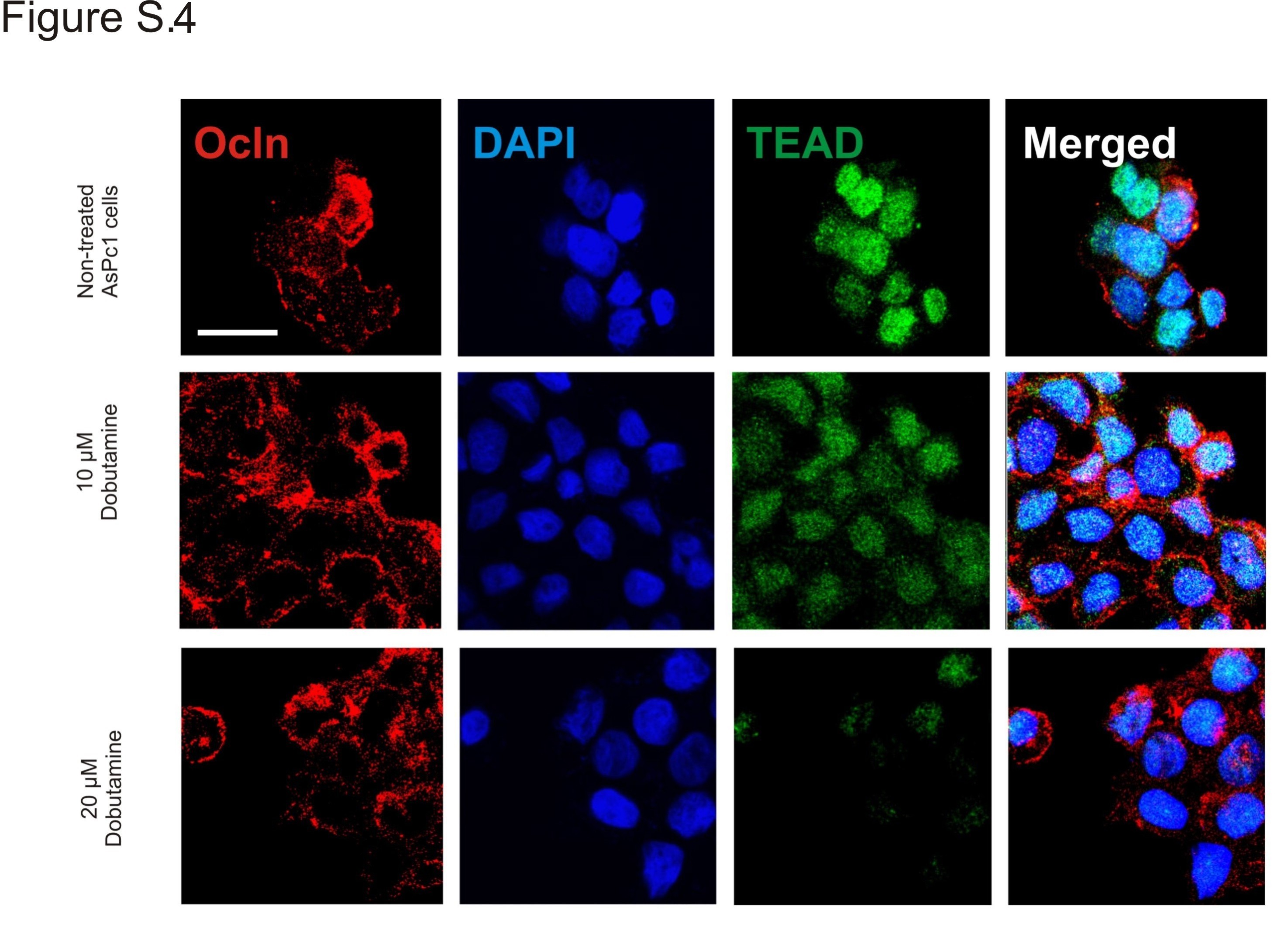

Supplement: Supplemental data for this article can be accessed on the publisher's website. [file ktib-03-03-1037948-s001.zip › Supplemental Material/Supplemental Figure 4.jpg]

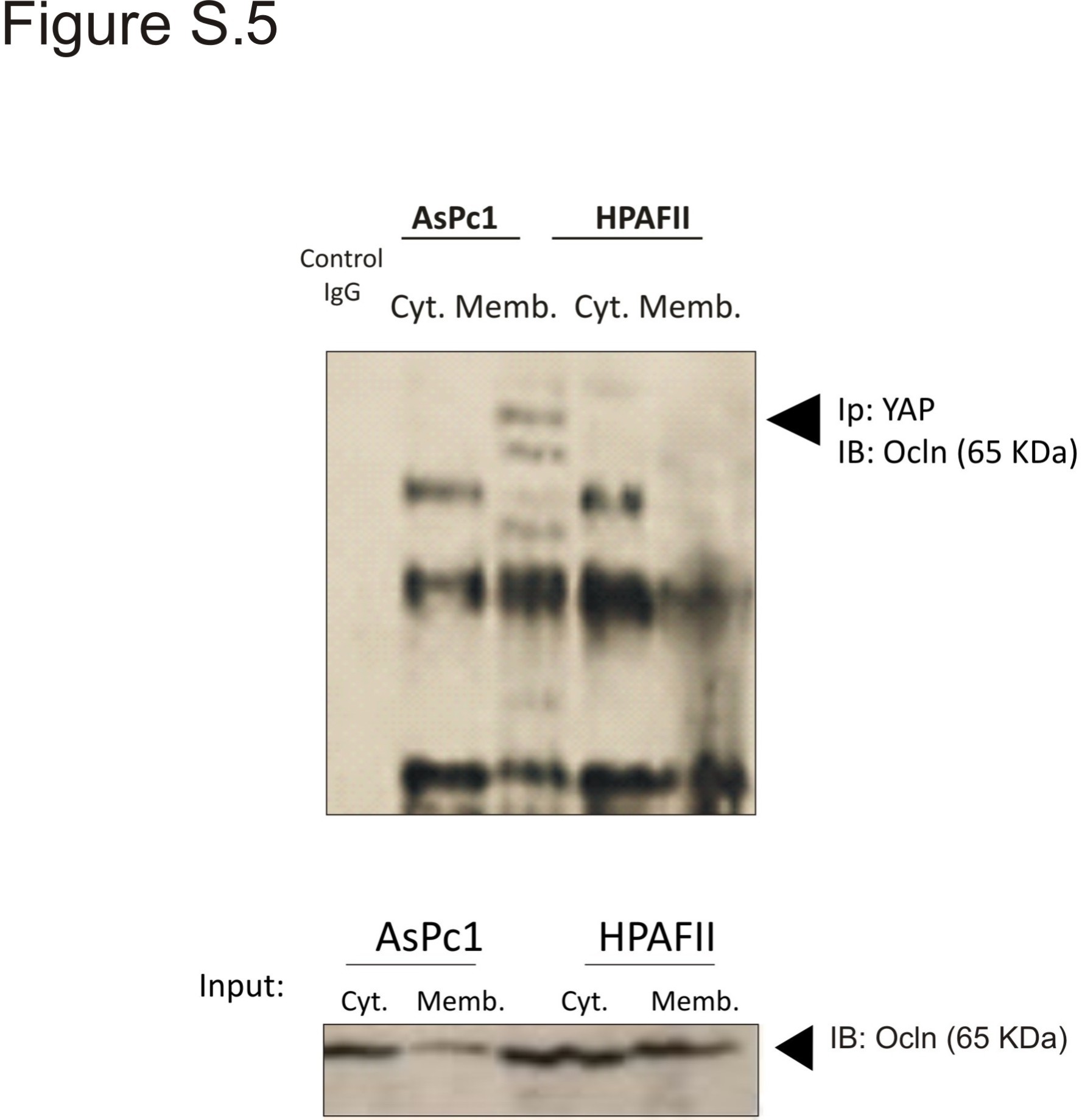

Supplement: Supplemental data for this article can be accessed on the publisher's website. [file ktib-03-03-1037948-s001.zip › Supplemental Material/Supplemental Figure 5.jpg]

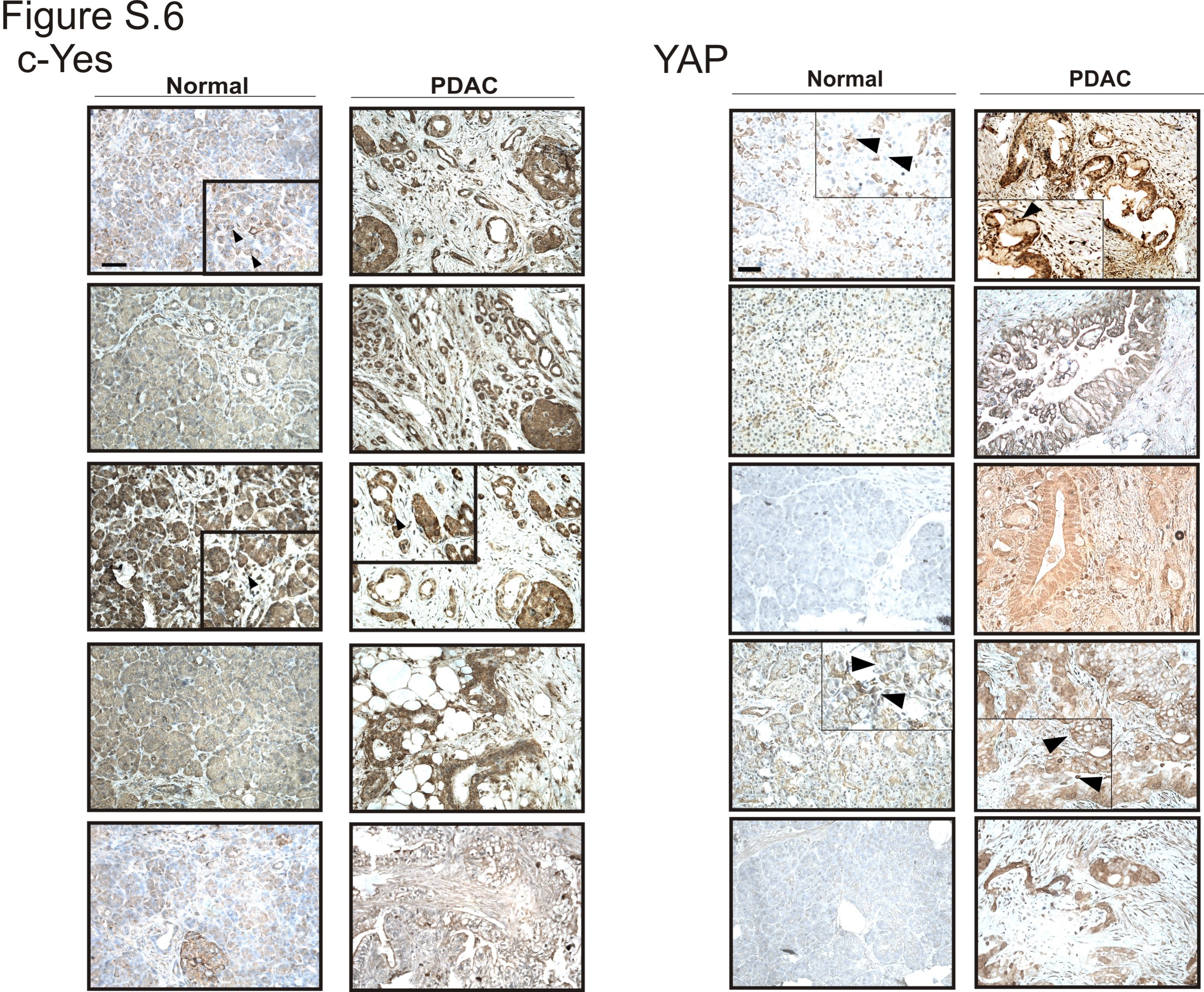

Supplement: Supplemental data for this article can be accessed on the publisher's website. [file ktib-03-03-1037948-s001.zip › Supplemental Material/Supplemental Figure 6.jpg]

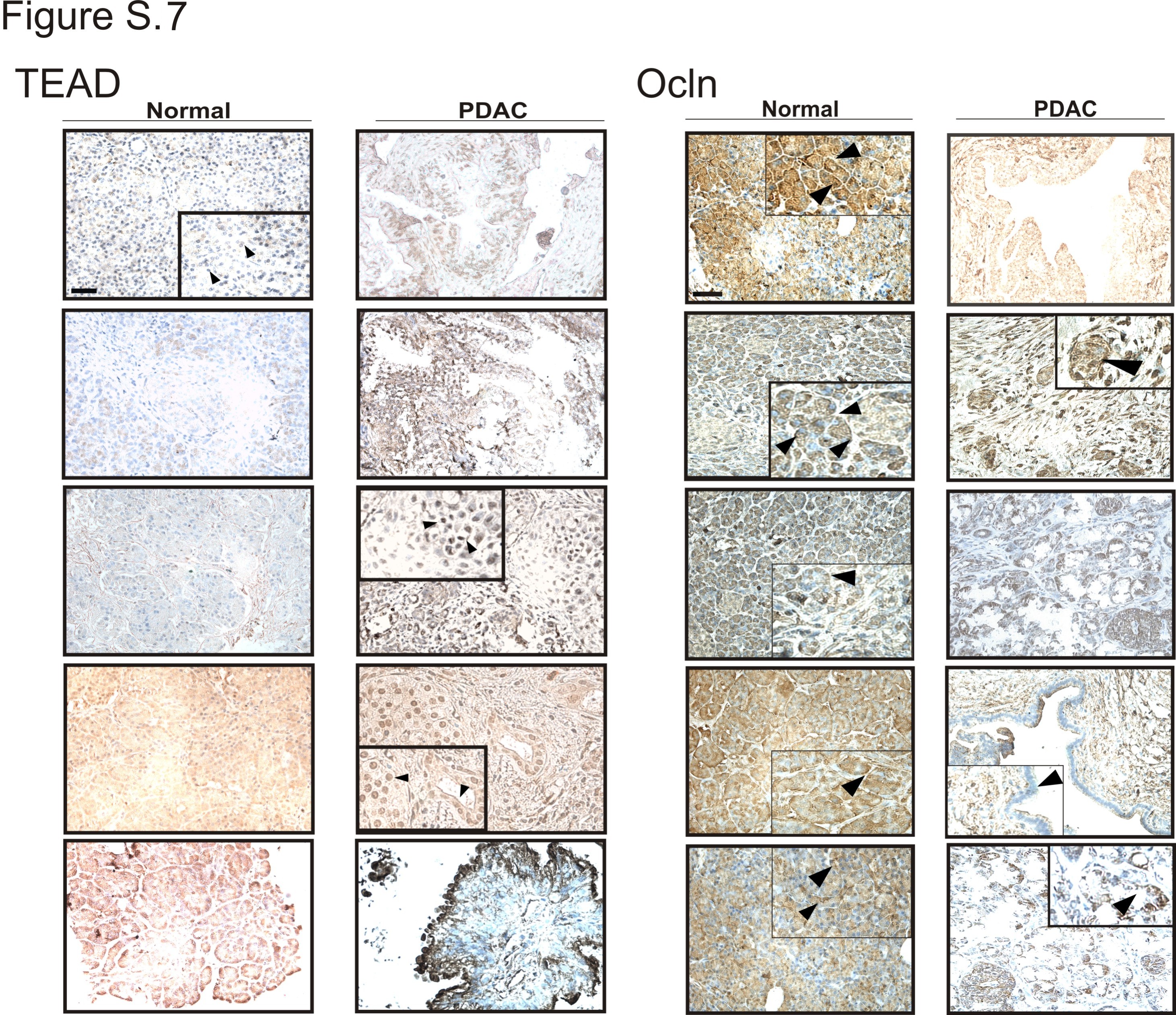

Supplement: Supplemental data for this article can be accessed on the publisher's website. [file ktib-03-03-1037948-s001.zip › Supplemental Material/Supplemental Figure 7.jpg]
